# The policy implemented by the government and the protection effect of PM2.5 decreasing on blood pressure in adolescents: From a quasi-experimental study

**DOI:** 10.7189/jogh.13.04050

**Published:** 2023-05-26

**Authors:** Xiaohua Liang, Fengchao Liang, Fangchao Liu, Yanling Ren, Jishuang Tong, Wei Feng, Ping Qu, Shunqing Luo

**Affiliations:** 1 Department of Clinical Epidemiology and Biostatistics, Children’s Hospital of Chongqing Medical University, National Clinical Research Center for Child Health and Disorders, Ministry of Education Key Laboratory of Child Development and Disorders, Chongqing Key Laboratory of Pediatrics, Chongqing, China; 2 School of Public Health and Emergency Management, Southern University of Science and Technology, Shenzhen, China; 3 Department of Epidemiology, Fuwai Hospital, National Center for Cardiovascular Diseases, Chinese Academy of Medical Sciences and Peking Union Medical College, Beijing, China

## Abstract

**Background:**

High particulate matter with an aerodynamic diameter of 2.5 μm or less (PM2.5) exposure levels posed a great risk to human health, but the protection effects of environmental protection on cardiovascular disease have not been systematically evaluated. This study aims to illustrate the effect of the decreased concentration of PM2.5 on blood pressure level in adolescents after enacting the protection measures of environment from a cohort study.

**Methods:**

A quasi-experimental study including 2415 children from the Chongqing Children’s Health Cohort, aged 7.32 ± 0.60 years with normal blood pressure at baseline, with 53.94% males, were analysed. Both the generalised linear regression model (GLM) and Poisson regression model were used to calculate the impact of the declining exposure level of PM2.5 on blood pressure and the incidence of prehypertension and hypertension.

**Results:**

The annual mean PM2.5 concentration in 2014 and in 2019 were 65.01 ± 6.46 µgmes per cubic metre (μg / m^3^), 42.08 ± 2.04 μg / m^3^ respectively, and the decreased PM2.5 concentration between 2014 and 2019 was 22.92 ± 4.51 μg / m^3^. The effect of decreased PM2.5 concentration by 1μg / m^3^ on systolic blood pressure (SBP), diastolic blood pressure (DBP), mean arterial pressure (MAP) and the difference of blood pressure (BP) indexes between 2014 and 2019 were all significant (*P* < 0.001). The absolute differences of SBP (-3.598 mmHg; 95% confidence interval (CI) = -4.47,-2.72 mm Hg), DBP (-2.052 mmHg; 95% CI = -2.80,-1.31 mm Hg) and MAP (-2.568 mmHg; 95% CI = -3.27,-1.87 mm Hg) in the group with a decreased level of ≥25.56 μg / m^3^ were more significant than those in a decreased concentration of PM2.5 for <25.56 μg / m^3^ (*P* < 0.001). And the incidence of prehypertension and hypertension for three occasions blood pressure diagnose was 2.21% (95% CI = 1.37%-3.05%, *P* = 0.001) in children with PM2.5 decreased level ≤25.56 μg / m^3^ (50%), which was significant higher than its’ counterparts 0.89% (95% CI = 0.37%-1.42%, *P* = 0.001).

**Conclusions:**

Our study found the etiological relationship between the declining PM2.5 concentration and the B*P* values and the incidence of prehypertension and hypertension in children and adolescents, suggesting continuous environmental protection measures in China have achieved remarkable health benefits.

Hypertension is one of the most important contributors to a severe disease burden worldwide; it is the leading risk factor in terms of cardiovascular disease (CVD) [1]. Hypertension in childhood has a global prevalence of approximately 4.0% and is significantly higher in low- and middle-income developing countries than in developed countries [2]. It is also the most common CVD in children. Childhood hypertension is an important risk factor for the development of adult hypertension, which is closely related to the development of cardiovascular disease in adulthood [3] and it is an independent impact factor of adult cardiovascular-related mortality [4]. Thus, identifying modifiable risk factors for childhood hypertension is a research priority.

Hypertension has well-established associations with lifestyle and genetics [5,6]. In addition, environmental pollutants also contribute to the occurrence of hypertension [7]. Recent studies have illustrated that exposure to air pollutants increased cardiovascular events and mortality [8,9]. Air pollution is the most important environmental health risk factor according to the World Health Organization (WHO), with fine particulate matter (PM2.5) being one of the most important pollutants [10]. Previous studies have indicated that for individuals exposed to ambient PM2.5 within several hours to days, their arterial blood pressure value would elevate [11]. In particular, it has been reported that PM2.5 might be a precursor of elevated blood pressure (BP) and the incidence of hypertension [12,13].

In the past half-century, along with rapid economic development, a number of countries have been transformed into industrial-based urban economies [14]. As a consequence, numerous necessary social productive activities emit harmful particulates (defined as PM10 / PM2.5) and gases (ie, ozone, nitrogen dioxide), and thus the air quality has deteriorated [15]. As in developing countries, such as China, large population and rapidly economic growth have posed an increased pressure to reduce the environment pollution [16]. WHO indicated that poor air quality causes over seven million premature deaths globally every year [17], especially in developing countries [18]. The air pollutants not only have a detrimental impact on human health but also subsequently degrade life satisfaction [19]. Moreover, many countries have recognized that environmental pollution is a vital risk factor for public health. Therefore, policies and rules were enacted to improve the environment pollution in developed countries, and the air quality has already been improved [20]. Therefore, in order to curb the excessive destroy of environment and to achieve the health promotion of environmental protection, the government of China has adopted various environmental protection measures to reduce the emission of pollutants and remove heavily polluting industries out of the city centre [21]. The government of China implemented several policies from 2012 to 2016 to reduce the environment pollution (**Table 1**). A study, estimation of ground-level PM2.5 concentration using MODIS AOD and a corrected regression model in Beijing, found that annual average PM2.5 concentration decreased by about 17% [22]. The result confirmed the effectiveness of the implementation of the toughest clean air policy by the Chinese government from 2013 to 2017 [23]. However, the health effect of environment protection is unclear.

**Table 1:** Policy implemented by the government of China from 2012 to 2016

Years	Policy implemented by the government of China
**2012**	In 2012, the government began monitoring haze pollution in various regions according to China's Ministry of Environmental Protection (MEP) adopted the Ambient Air Quality Standards (DEP, 2012).
**2013**	The Action Plan for Prevention and Control of Air Pollution (China State Council, 2013) specified haze control measures and set clear air pollution control targets for each scope.
**2013**	In response to severe air pollution, the Chinese government's 12th Five-Year Plan (Ministry of Environmental Protection of China, 2011) for sulphur dioxide and nitrogen oxide control, and an action plan for air pollution prevention and control were implemented in 2013 (State Council, China, 2013)
**2016**	In order to promote an ecologically sustainable society, Chinese government enacted the first law to implement an environmental protection tax (the Environmental Protection Tax Law of the People's Republic of China (EPTL 2016))

Previous studies mainly explored the dose-relationship between PM2.5 exposure and BP level or hypertension [24-26]. However, limited studies illustrate the positive health protective effect of PM2.5 decreasing. Until recently, several studies have indicated the potential benefits of indoor use of air filters on cardiopulmonary health [27,28]. These studies were mostly conducted in less polluted countries (with lower PM2.5 concentration than China), and mainly focused on indoor pollutants improvement rather than on natural PM2.5 concentration decrease, which did not represent the health effects of natural air pollution quality improvement on blood pressure in China. Evidence from animals found in spontaneously hypertensive rats [29] exposed to PM2.5 showed that BP reversibly recovered when exposure to PM2.5 was stopped [30]. When the exposure level of air pollutants in environment decreased, BP of human beings would be improved [27,28,30]. In addition, there was a lack of research on the protective effect of declining PM2.5 on hypertension, especially on children and adolescents, whose hypertension is more likely to be prevented and controlled by external interventions [31]. Therefore, the hypothesis of this study is that the declining concentration of outdoor PM2.5 will have a protection effect on BP value or the incidence of hypertension in children. And this study will conduct a well-designed large sample size prospective cohort study to explore the protective effect of declining PM2.5 exposure on BP in children and adolescents.

## METHODS

### Participants

**Figure 1:**
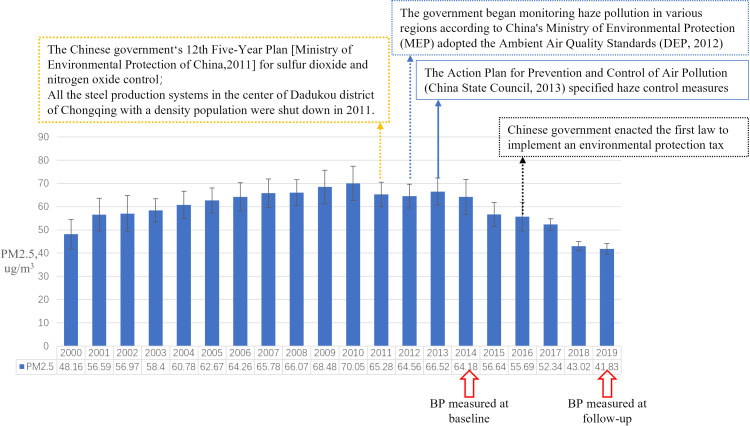
The relationship between policies and measures for the prevention and control of particulate matter with an aerodynamic diameter of 2.5 μm or less (PM2.5).

The subjects were from the Chongqing Children’s Health Cohort (CCHC). A two-stage stratified cluster sampling was used to recruit subjects, and the recruitment flow was described in our previous publications [17,32,33]. Briefly, one urban county and one rural county were randomly chosen from Chongqing, which may represent the urban-rural areas. Then, one community in rural county and urban county was chosen, and all the primary school in the community were included. And the children in grade one and grade two were included at baseline. And the inclusion criteria was as follows: (1) children were 6-8 years old at baseline (in 2014); (2) living in the chosen areas for >6 months; (3) without prehypertension or hypertension for the first occasion BP measurement at first visit; (4) home address was recorded at two visits;(5) without serious disease (such as congenital heart disease, kidney disease, or cancer, et al.); (6) both in 2014 and in 2019, children and their parents or guardians signed informed consents to participate in the cohort study. This study used the data of two visits, as the physical measurement in 2014 (base on three-time BP measurements at three occasions) was used to exclude the children with prehypertension or hypertension, and the BP measurement in 2019 was used to diagnose adolescents with new onset prehypertension or hypertension. Moreover, the difference of BP levels from baseline in 2014 to the follow-up in 2019 were calculated. At baseline, questionnaires were used to select participants who were qualify inclusion criteria. Individual PM2.5 exposure level was used to evaluate the pollution status of participants, and the decreased level of PM2.5 from 2014 to 2019 (**Figure 1** and Table S11 in the **Online Supplementary Document**) was considered as a protective factor of both B*P* values in 2019 and the difference of BP levels from baseline in 2014 to the follow-up in 2019.There were 3866 children with home address and physical measurement; 574 samples were excluded as they entered this cohort in 2016 or in 2019, which was not at baseline in 2014. Finally, 2415 participants with normal BP at baseline were included (Figure S1 in the **Online Supplementary Document**). This study was carried out under the agreement of the Institutional Review Board of Children's Hospital of Chongqing Medical University (No. 2019-86).

### Physical examination

The demographic characteristics and physical measurements were surveyed at two visits (the baseline visit and the follow-up visit). Well trained paediatric nurses and doctors joined in the physical examination (height, weight, waist circumference and BP) and the detail protocol was published in previous papers [17,32,34,35]. Briefly, BP was measured using an arm-type electronic sphygmomanometer (OMRON, HEM7051), and an appropriately sized BP cuff, placed on the right arm of the children, with a seated position, which was detailed in our previous published papers [32,36-38]. During a 15-minute relaxation period (8:30-12:00 am), BP was measured at three time points, at the 11, 13, and 15 minutes respectively. Then, the average systolic blood pressure (SBP) and diastolic blood pressure (DBP) values were calculated using all three time points’ BP levels. If the children were diagnosed as prehypertension or hypertension only using B*P* values, secondary hypertension was also checked by doctors through reviewing participants’ disease symptoms (such as oedema, headache, and dizziness, etc.), measuring the heart and kidneys by ultrasonography and visible parameters, checking biochemical indexes. Mean arterial pressure [39] was calculated as mean arterial pressure (MAP) = (SBP + 2 × DBP) / 3.

### Case definitions

Three separate occasions BP measurements, with one week interval, were used to diagnose the new onset prehypertension or hypertension if all three average SBP or DB*P* values met the diagnose criteria from China (Table S1 in the **Online Supplementary Document**) [40]. Prehypertension and hypertension were defined based on age, sex and height specific percentiles of SBP and / or DB*P* values≥P_90_ ~ <P_95_, and hypertension was diagnosed based on SBP and / or DB*P* values≥P_95_. Moreover, systolic or diastolic prehypertension and hypertension were also diagnosed by the above criteria, if SBP or DBP met the age, sex and height specific percentiles of SBP or DB*P* values.

### Demographic characteristics

The demographic variables were collected both at baseline and at follow-up using a self-filled structured questionnaire, which surveyed perinatal variables about maternal obesity and birthweight, maternal education, puberty development, passive smoking and household income using a structured questionnaire form, as described in our published papers [37]. The data was provided both by the children and their guardian following a detail protocol, which introduced the method to fill out the questionnaire form. Briefly, passive smoking (PS) of children was investigated by a questionnaire, which included the exposed frequency and dose during perinatal, at childhood and adolescent stage. The PS questionnaire were answered by mothers, who may provide the truth exposure level, and the calculated flow was introduced in our papers. [17] The levels of physical activity (PA) for participants at the follow-up visit were surveyed and analysed in this study. [41] Moreover, puberty development was surveyed by pedestrians. They were asked about growth and development status. Besides that, a self-filled questionnaire was answered by children and their guardians to ensure the accuracy of the puberty development. Sleep quality of participants was investigated using the Children’s Sleep Habits Questionnaire (CSHQ) [42]. In addition, a quantitative dietary questionnaire, including the frequency and dose of dietary intake, was used to collect dietary information, as it was described in former papers [43].

### Biochemical indexes

Venous blood was collected at 7:30-10:30 am, 12 hours after fasting, with serum and plasma separated from blood, which was introduced by our previous publications [17,33,34]. Then, the serum samples were kept in a -80°C refrigerator for the later biochemical measurement. Glucolipid metabolism indexes were checked by an automatic biochemical analyser (Mindray BS-800), which included serum fasting blood glucose (FBG), total cholesterol (TC), triglycerides (TG), high-density lipoprotein cholesterol (HDL-C), low-density lipoprotein cholesterol (LDL-C). In addition, serum level of renal function indexes of uric acid (UA) and creatinine were measured.

### The policies and measures for the prevention and control of PM2.5 in Chongqing

A quasi-experimental study was used to analyse the effect of environmental protection on blood pressure levels. In order to realize the target of environmental protection, several effective actions were implemented in Chongqing (**Figure 1**). First, the government enacted policies to control haze and to keep clean energy projects. Recently, the government focused on the prevention and control of air pollutants in the main urban area and has implemented clean energy projects in Chongqing. As the five-pronged “clearance projects”, “blue sky action” in the main city and other measures were also effectively executed; at the same time, measures were taken to promote the construction of clean fuel, smoke and dust control region. Basically, coal-free areas have been implemented in all regions, and the quality of air has been improved year by year. For example, owing to environmental relocation, all the steel production systems in the centre of Dadukou district with a density population were shut down in 2011. The government vigorously has been developing public transport and restricting the number of private cars using oil. In the past five years, the main urban region of Chongqing has achieved full coverage of subways and buses, which is convenient for urban residents to use public transportation. By rationally arranging traffic and routes, reducing congestion, controlling the number of private cars, and restricting the number of vehicles using fuel on weekdays, the exhaust emissions of motor vehicles were also reduced. Also, a comprehensive environmental management and prevention of the emission of polluting gases were strengthened. In the urban area, the government comprehensively prohibits outdoor and indoor smoking bacon, open burning, and other behaviours that pollute the atmospheric environment; the government stops all barbecue and firewood turkey catering operators from using high-polluting fuels and suggests those companies to switch to work with natural gas, electricity, liquefied petroleum gas or other clean energies. In the rural area, the government encourages farmers to use natural gas as fuel instead of burning wheat straw and prohibits farmers to burn wheat straw in the fall harvest season. In addition, Chongqing is striving to build a city with beautiful mountains and rivers. Through the continuous implementation of the “Five Actions” of blue sky, clear water, green space, tranquillity, and pastoral gardens, a total of more than 150 billion yuan has been invested to control pollution and improve the ecological conditions in the past years. The main policy was started from 2011 to 2016, which has been implementing continuously from then on.

### Evaluation of the exposure to PM2.5

Machine learning was used to compute the monthly mean PM2.5 exposure level from perinatal to adolescent of each participant. The method with aim of validating the spatial resolution of PM2.5 calculated to one km, has been introduced in our previous papers. [17,44,45]. Machine-learning approaches were used to calculate the monthly mean PM 2.5 concentrations in the Chongqing urban-rural areas from 2005 to 2019. Moreover, the Multiangle Implementation of Atmospheric Correction AOD product made available by NASA was used to enhance the spatial resolution of PM2.5 estimates to one km, and this method was described in a previous study. Moreover, the home addresses of each subject from pregnancy to the two visits (in 2014 and in 2019), from 2005 to 2019 were geocoded, and the monthly mean value of PM2.5 was assigned to each participant. If children moved their home address during the follow-up period, the monthly mean value of PM2.5 at the addresses before and after the movement was calculated. In addition, the school addresses were collected, and the monthly average PM2.5 concentration around the school were also computed. Then, the annual mean value of PM2.5 was computed by the following steps [17]. First, the annual mean values of PM2.5 of home address (HPM2.5) and school address (SPM2.5) were computed using the weighted arithmetic means of monthly mean value of the corresponding year. Second, the annual mean values of PM2.5 of each participants combined home address and school address (HSPM2.5), were calculated as HSPM2.5 = 2 × HPM2.5 / 3 + SPM2.5 / 3, as children spend two thirds of the time at home and one third in school. Third step, each child exposed to PM2.5 concentration in 2014 (65.01 ± 6.46 μg / m^3^) and in 2019 (42.08 ± 2.04 μg / m^3^), and the decreased level from 2014 to 2019 (22.92 ± 4.51μg / m^3^) were used to represent the decreased PM2.5 concentration (**Table 1**). And the decreased individual level of PM2.5 exposure = PM2.5 concentration in 2014 (a high level) – PM2.5 concentration in 2019 (a decreased status). The decreased PM2.5 concentration of each subject was computed as HSPM2.5 concentration in 2014 minus HSPM2.5 concentration in 2019.

### Statistical analyses

Social economic status (SES) variables and anthropometric indexes with normal distribution, were expressed as mean ± STD, and categorical variables was indicated as n (%). General linear regression model (GLM) was used to analyses the impact of long-term (from 2014 to 2019) decreased individual PM2.5 exposure (as independent variable) on BP levels in 2019 or the decreased BP levels between the follow-up and baseline (as dependent variable), which was analysed using four models. Model one was a crude model, and model two adjusted age, sex, height and weight. Multiple variables of maternal education, puberty development, passive smoking, maternal obesity, birthweight, household income, vegetable intake, red meat intake, pickle intake, sleep quality and physical activity, were added to model two, which formed model three. Then, biochemical indexes of FBG, insulin, creatinine, TG and HDL were added to model three, which formed model four. In addition, the difference of B*P* values between children with decreased concentration of PM2.5≤P_50_ (a high exposure group) and those with decreased concentration of PM2.5>P_50_ were analysed by covariance analysis using four models to adjust covariates. The incidence of prehypertension and hypertension for three occasions BP measurements were calculated by a Poisson regression model (using “Proc Genmod” in SAS). In addition, the difference of the incidence rate between children with decreased concentration of PM2.5≤P_50_ (a high exposure group) and those with decreased concentration of PM2.5>P_50,_ were computed using “Genmod” and “% NL Means” command of SAS. Moreover, the risk rate (RR) of prehypertension and hypertension between two exposure groups was calculated by Poisson regression model using four model to adjust covariates.

The sensitivity analyses were made to deal with the non-independence within each cluster, and a linear mixed model was used to adjust the cluster effect of the non-independence within urban or rural cluster. And four models were made to adjusted the covariates which may impact the effect of BP levels as aforementioned.

All the analyses in this study were made by SAS 9.4 software (Copyright © 2016). The statistical difference was defined by an α level of 0.05, using a two-sided test.

## RESULTS

### General characteristics

**Figure 2:**
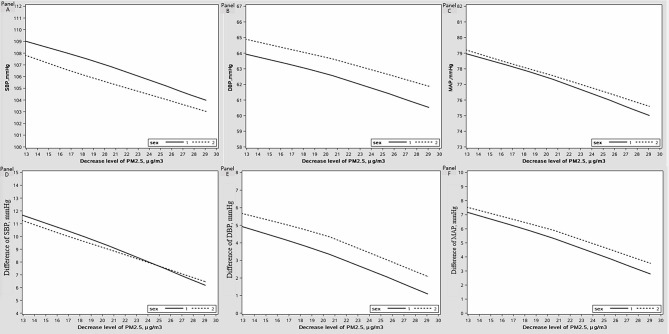
The relationship between decreased level of blood pressure (BP) levels and the decreased particulate matter with an aerodynamic diameter of 2.5 μm or less (PM2.5) value.

The demographic characteristics of the participants at baseline and follow-up were presented in **Table 2**. At baseline, the average age of participants was 7.32 ± 0.60 years old with an annual mean PM2.5 exposure concentration of 65.01 ± 6.46 μg / m^3^, and the annual mean PM2.5 concentration in 2019 were 42.08 ± 2.04 μg / m^3^. Among all participants, 53.50% (1292 / 2415) were males. The decreased level of PM2.5 from 2014 to 2019 was 22.92 ± 4.51 μg / m^3^. The hemodynamic indexes (ie, SBP, DBP and MAP) and the difference of hemodynamic indexes between follow-up and baseline was shown in **Table 1**. The difference in decreased exposure level of PM2.5 from 2014 to 2019 in male and female was shown in **Figure 1**, and the relationships between decreased exposure level of PM2.5 and BP levels was shown in **Figure 2** from panel A to panel F. The level of SBP and the decreased level of SBP were greater in male accompanied with the decreased PM2.5 value (**Figure 2**, panel A and panel D). Moreover, other anthropometric indicators were shown in **Table 2**.

**Table 2:** The characteristics of children at baseline by decreased PM2.5<P_50_ (25.56 μg / m^3^)

Variables	Total	Decreased PM2.5 level <25.56 μg / m^3^	Decreased PM2.5 level ≥25.56 μg / m^3^	*P*-value
At baseline in 2014				
*Birthweight, g*	3239.4 ± 525.50	3241.4 ± 532.80	3237.4 ± 518.70	0.853
*Age, years*	7.32 ± 0.60	7.33 ± 0.62	7.31 ± 0.57	0.304
*Waist, cm*	54.35 ± 6.37	52.79 ± 5.63	55.85 ± 6.68	<0.001
*Height, cm*	124.01 ± 6.13	122.89 ± 5.92	125.08 ± 6.14	<0.001
*Weight, kg*	25.04 ± 4.95	24.06 ± 4.67	25.98 ± 5.02	<0.001
*SBP, mmHg*	97.11 ± 6.69	97.01 ± 6.73	97.21 ± 6.65	0.464
*DBP, mmHg*	59.37 ± 5.30	59.18 ± 5.39	59.55 ± 5.21	0.088
*MAP, mmHg*	71.95 ± 5.07	71.79 ± 5.14	72.10 ± 4.99	0.130
*Heart rate, n / min*	96.70 ± 11.66	96.52 ± 11.76	96.88 ± 11.56	0.458
*Creatinine, mmol / L*	54.04 ± 21.89	54.26 ± 23.14	53.94 ± 21.31	0.824
*Triglyceride, mmol / L*	0.94 ± 0.56	0.89 ± 0.60	0.95 ± 0.54	0.106
*HDL, mmol / L*	1.27 ± 0.27	1.33 ± 0.28	1.25 ± 0.27	<0.001
At follow-up in 2019				
*Age, years*	11.77 ± 0.62	11.86 ± 0.65	11.68 ± 0.58	<0.001
*Waist, cm*	64.25 ± 9.74	62.07 ± 9.51	66.35 ± 9.49	<0.001
*Height, cm*	151.58 ± 7.89	150.92 ± 7.71	152.21 ± 8.01	<0.001
*Weight, kg*	43.35 ± 10.12	42.94 ± 9.97	43.75 ± 10.24	0.047
*SBP, mmHg*	105.44 ± 9.17	107.06 ± 9.62	103.90 ± 8.43	<0.001
*DBP, mmHg*	62.51 ± 6.66	63.53 ± 7.04	61.54 ± 6.11	<0.001
*MAP, mmHg*	76.82 ± 6.76	78.04 ± 7.20	75.66 ± 6.10	<0.001
*Heart rate, n / min*	88.17 ± 11.77	86.74 ± 11.61	89.52 ± 11.77	<0.001
*Creatinine, mmol / L*	53.03 ± 19.35	53.21 ± 23.55	52.85 ± 14.23	0.652
*Triglyceride, mmol / L*	1.03 ± 0.55	1.00 ± 0.61	1.07 ± 0.47	0.003
*HDL, mmol / L*	1.44 ± 0.31	1.43 ± 0.31	1.44 ± 0.30	0.188
*FBG, mmol / L*	4.39 ± 0.43	4.27 ± 0.40	4.50 ± 0.43	<0.001
*Insulin, IU*	78.83 ± 88.14	68.69 ± 83.51	88.54 ± 91.33	<0.001
Physical activity, min / day	103.22 ± 80.92	101.81 ± 84.63	104.58 ± 77.23	0.401
Sleep score	45.71 ± 6.39	46.10 ± 6.67	45.35 ± 6.10	0.004
Dietary intake				
*Vegetables intake, g / day*	202.86 ± 193.90	158.20 ± 176.90	245.61 ± 199.80	<0.001
*Red meat intake, g / day*	145.16 ± 196.30	134.81 ± 202.60	155.06 ± 189.70	0.011
*Cereals and fruit, g / day*	11.98 ± 19.89	13.44 ± 23.18	10.58 ± 16.01	<0.001
Sex, male, n (%)	1292 (53.50)	637 (53.94)	655 (53.08)	0.673
Puberty development, n (%)	562 (23.27)	270 (22.86)	292 (23.66)	0.642
Passive smoking, n (%)	1000 (41.41)	404 (34.21)	596 (48.30)	<0.001
Maternal obesity	216 (8.94)	104 (8.81)	112 (9.08)	0.816
Income, Yuan, n (%)				
*~ 500*	187 (7.74)	126 (10.67)	61 (4.94)	<0.001
*~ 1000*	285 (11.80)	184 (15.58)	101 (8.18)	
*~ 2000*	468 (19.38)	278 (23.54)	190 (15.40)	
*~ 3000*	488 (20.21)	240 (20.32)	248 (20.10)	
*>3000*	987 (40.87)	353 (29.89)	634 (51.38)	
Personality				
*Introvert type*	331 (13.71)	185 (15.66)	146 (11.83)	0.002
*Intermediate type*	800 (33.13)	357 (30.23)	443 (35.90)	
*Extrovert type*	1284 (53.17)	639 (54.11)	645 (52.27)	
The difference between follow-up and baseline				
*Different of SBP, mmHg*	8.33 ± 9.63	10.05 ± 10.95	6.70 ± 7.83	<0.001
*Different of DBP, mmHg*	3.14 ± 7.76	4.35 ± 8.38	1.99 ± 6.93	<0.001
*Different of MAP, mmHg*	4.87 ± 7.46	6.25 ± 8.26	3.56 ± 6.33	<0.001
*Different of Heart rate*	-8.53 ± 13.71	-9.76 ± 13.83	-7.35 ± 13.51	<0.001
PM2.5 exposure level, μg / m^3^				
*In 2014*	65.01 ± 6.46	59.07 ± 3.95	70.69 ± 0.75	<0.001
*In 2019*	42.08 ± 2.04	40.24 ± 1.30	43.85 ± 0.33	<0.001
*Difference of PM2.5 level*	22.92 ± 4.51	18.83 ± 2.84	26.84 ± 0.80	<0.001

PM2.5 – particulate matter with an aerodynamic diameter of 2.5 μm or less, μg / m^3^ – microgrammes per cubic metre, g – grammes, cm – centimetres, kg – kilogrammes, SBP – systolic blood pressure, mmHg – millimetre of mercury, DBP – diastolic blood pressure, MAP – mean arterial pressure, HDL – high-density lipoprotein, FBG – fasting blood-glucose, min – minute, mmol / L – millimoles per litre, IU – international unit

### The effect of the declining PM2.5 concentration on BP values

The results of the impact of decreased PM2.5 levels on B*P* values were indicated in **Table 3** and Table S2 in the **Online Supplementary Document**. The decreased level of PM 2.5 by 1μg / m^3^ was negatively associated with SBP (β = -0.303; 95% confidence interval (CI) = -0.384,-0.223, *P* < 0.001), DBP (β = -0.206; 95% CI = -0.264,-0.147, *P* < 0.001), and MAP (β = -0.238; 95% CI = -0.297,-0.179, *P* < 0.001) in model one. After adjusted age, sex, height, weight, maternal education, puberty development, passive smoking, maternal obesity, birth weight, household income, vegetable intake, red meat intake, pickle intake, sleep quality, physical activity, personality character, FBG, insulin, creatinine, TG and HDL in model four, three hemodynamic indexes also statistically correlated with the decreased level of PM2.5 by 1 μg / m^3^ (all *P* < 0.001). In addition, the net difference of blood pressure values in 2019 compared with these values in 2014, the decreased level of PM2.5 by 1 μg / m^3^ was also negatively associated with the net difference in SBP (β = -0.376; 95% CI = -0.452,-0.299, *P* < 0.001), DBP (β = -0.216; 95% CI = -0.279,-0.153, *P* < 0.001), and MAP (β = -0.269; 95% CI = -0.330,-0.208, *P* < 0.001) in model four after adjusted covariates (*P* < 0.001) (**Table 3**).

**Table 3:** The effect of 1 μg / m^3^ decrease of the PM2.5 on blood pressure value

	Model 1*		Model 2†		Model 3‡		Model 4§	
**Dependent variables**	**β (95% CI)**	***P*-value**	**β (95% CI)**	***P-*value**	**β (95% CI)**	***P*-value**	**β (95% CI)**	***P*-value**
BP value								
*SBP, mmHg*	-0.303 (-0.384,-0.223)	<0.001	-0.338 (-0.408,-0.269)	<0.001	-0.343 (-0.417,-0.270)	<0.001	-0.376 (-0.452,-0.299)	<0.001
*DBP, mmHg*	-0.206 (-0.264,-0.147)	<0.001	-0.199 (-0.257,-0.142)	<0.001	-0.206 (-0.267,-0.145)	<0.001	-0.216 (-0.279,-0.153)	<0.001
*MAP, mmHg*	-0.238 (-0.297,-0.179)	<0.001	-0.246 (-0.301,-0.191)	<0.001	-0.252 (-0.310,-0.193)	<0.001	-0.269 (-0.330,-0.208)	<0.001
The difference of BP values								
*Different of SBP, mmHg*	-0.321 (-0.405,-0.236)	<0.001	-0.341 (-0.422,-0.259)	<0.001	-0.315 (-0.402,-0.227)	<0.001	-0.345 (-0.435,-0.254)	<0.001
*Different of DBP, mmHg*	-0.241 (-0.310,-0.173)	<0.001	-0.226 (-0.294,-0.158)	<0.001	-0.203 (-0.277,-0.130)	<0.001	-0.207 (-0.284,-0.131)	<0.001
*Different of MAP, mmHg*	-0.268 (-0.333,-0.203)	<0.001	-0.264 (-0.329,-0.200)	<0.001	-0.240 (-0.310,-0.171)	<0.001	-0.253 (-0.325,-0.181)	<0.001

μg / m^3^ – microgrammes per cubic metre, PM2.5 – particulate matter with an aerodynamic diameter of 2.5 μm or less, CI – confidence interval, BP – blood pressure, SBP – systolic blood pressure, mmHg – millimetre of mercury, DBP – diastolic blood pressure, MAP – mean arterial pressure

*The crude model.

†Adjusted age, sex, height and weight.

‡Variables in Model 2 added maternal education, puberty development, passive smoking, maternal obesity, birthweight, household income, vegetable intake, red meat intake, pickle intake, sleep quality and physical activity.

§Variables in Model 3 added personality character, fasting blood-glucose (FBG), insulin, creatinine, triglycerides (TG) and high-density lipoprotein (HDL).

In addition, the impact of the decreased level of PM2.5 by P_50_ intervals (<25.56 μg / m^3^ and ≥25.56 μg / m^3^) on B*P* values was shown in **Table 4** using four models adjusted different covariates. Compared with decreased level of PM2.5 < 25.56 μg / m^3^, the difference in SBP (-3.538 mmHg; 95%CI = -4.29,-2.79 mmHg, *P* < 0.001), DBP (-1.860 mmHg; 95% CI = -2.49,-1.23 mm Hg, *P* < 0.001), and MAP (-2.420; 95% CI = -3.02,-1.82 mm Hg, *P* < 0.001) in those children with decreased level of PM2.5 ≥ 25.56 μg / m^3^ at follow-up were significant. The results showed that the net differences of SBP (-3.598 mmHg; 95% CI = -4.47,-2.72 mm Hg, *P* < 0.001), DBP (-2.052 mmHg; 95% CI = -2.80,-1.31 mm Hg, *P* < 0.001), and MAP (-2.568 mmHg; 95% CI = -3.27,-1.87 mm Hg, *P* < 0.001) were also significant compared the decreased level of PM2.5 ≥ 25.59 μg / m^3^ with <25.59 μg / m^3^ (Table S2 in the **Online Supplementary Document**). Moreover, the relationship was consistently statistically significant even when adjusting for variables in model four (*P* < 0.001).

**Table 4:** The impact of decreased PM2.5 by P_50_ (25.56 μg/m3) on the incidence of prehypertension and hypertension

Diagnosed of BP	Decreased level of PM2.5	Cases	Samples	Incidence (95% CI)	Difference of incidence (95% CI)	*P*-value
Prehypertension and hypertension						
*One occasion*						
	≤25.59 μg / m^3^	161	1178	13.67% (11.71%-15.63%)	4.02% (1.28%-6.75%)	0.004
	>25.59 μg / m^3^	119	1233	9.65% (8.00%-11.30%)		
*Two occasions*						
	≤25.59 μg / m^3^	54	1178	4.58% (3.39%-5.78%)	2.72% (1.28%-4.16%)	<0.001
	>25.59 μg / m^3^	23	1233	1.87% (1.11%-2.62%)		
*Three occasions*						
	≤25.59 μg / m^3^	26	1178	2.21% (1.37%-3.05%)	1.32% (0.37%-2.31%)	0.001
	>25.59 μg / m^3^	11	1233	0.89% (0.37%-1.42%)		
Systolic prehypertension and hypertension						
*One occasion*						
	≤25.59 μg / m^3^	123	1178	10.44% (8.70%-12.19%)	3.55%(1.19%-5.90%)	0.003
	>25.59 μg / m^3^	85	1233	6.89% (5.48%-8.31%)		
*Two occasions*						
	≤25.59 μg / m^3^	41	1178	3.48% (2.43%-4.53%)	2.26% (1.03%-3.49%)	<0.001
	>25.59 μg / m^3^	15	1233	1.22% (0.60%-1.83%)		
*Three occasions*						
	≤25.59 μg / m^3^	19	1178	1.61% (0.89%-2.33%)	0.72% (-0.18%,1.62%)	0.115
	>25.59μg/m3	11	1233	0.89% (0.37%-1.42%)		
Diastolic prehypertension and hypertension						
*One occasion*						
	≤25.59 μg / m^3^	71	1178	6.00% (4.67%-7.39%)	1.81% (-0.00%,3.62%)	0.050
	>25.59 μg / m^3^	52	1233	4.22% (3.10%-5.34%)		
*Two occasions*						
	≤25.59 μg / m^3^	29	1178	2.46% (1.58%-3.35%)	1.25% (0.16%-2.33%)	0.025
	>25.59 μg / m^3^	15	1233	1.22% (0.60%-1.83%)		
*Three occasions*						
	≤25.59 μg / m^3^	18	1178	1.53% (0.83%-2.23%)	1.12% (0.33%-1.91%)	0.005
	>25.59 μg / m^3^	5	1233	0.41% (0.05%-0.76%)		

PM2.5 – particulate matter with an aerodynamic diameter of 2.5 μm or less, μg / m^3^ – microgrammes per cubic metre, BP – blood pressure, CI – confidence interval

In addition, a sensitivity analysis was made to adjust the cluster effect of urban-rural areas (Table S3 in the **Online Supplementary Document**). The levels of SBP, DBP and MAP and the decreased levels of BP were significant after adjusted multivariable in model four.

### The impact of the decreased level of PM2.5 on prehypertension and hypertension

The effect of the decreased level of PM2.5 on the incidence rate (IR) of prehypertension and hypertension was indicated in **Table 4** and Table S4 in the **Online Supplementary Document**. The IR of prehypertension / hypertension for three occasions were 2.21% (26 / 1178) and 0.89% (11 / 1233) for children with the decreased PM2.5 level <25.59 μg / m^3^ or ≥25.59 μg / m^3^, respectively. In addition, compared the decreased level of PM2.5 < 25.59 μg / m^3^, the incidence of prehypertension / hypertension had significant difference even for three occasions (IR = 1.32%; 95% CI = 0.37%-2.31%, *P* = 0.001) (**Table 4**). Moreover, the incidence of systolic / diastolic prehypertension and hypertension for three different times’ measurement was also higher compared the decreased level of PM2.5 < 25.59 μg / m^3^ with those children with decreased level of PM2.5 ≥ 25.59 μg / m^3^. However, the IR of systolic prehypertension / hypertension (IR = 3.55%; 95% CI = 1.19%-5.90%, *P* = 0.003) and diastolic prehypertension/hypertension (IR = 1.81%; 95% CI = -0.00%-3.62%, *P* = 0.050) had the largest difference on the first occasion BP measurement.

Compared with the decreased level of PM2.5 < 25.59 μg / m^3^, the IR of prehypertension / hypertension decreased 59.6% with risk ratio (RR) = 0.404; 95% CI = 0.201-0.814, IR of systolic prehypertension / hypertension decreased 44.7% with RR = 0.553; 95% CI = 0.264-1.157 and IR of diastolic prehypertension / hypertension decreased 73.5% with RR = 0.265; 95% CI = 0.099-0.713, with the decreased level of PM2.5 ≥ 25.59 μg / m^3^. The relationships between the risk of hypertension / prehypertension and systolic / diastolic prehypertension / hypertension and the decreased level of PM2.5 were significant, after adjusted covariates (all *P* < 0.05) (Table S4 in the **Online Supplementary Document**).

## DISCUSSION

This study investigated the effect of declining ambient PM2.5 exposure concentration on BP levels in children and adolescents aged 6-13 years in Chongqing using a prospective cohort study. Improvements in air quality was observed from 2014 to 2019, with a significant decrease in PM2.5 concentrations. We found that a decrease of PM2.5 concentration had a protective effect on children's BP level, and the IR of prehypertension and hypertension. This study found a continual negative relationship between the B*P* values and decreased PM2.5 level. In addition, boys had higher SBP and lower DBP than girls accompanied with the decrease level of PM2.5.

This study illustrated the protective effect of air quality improvement on BP in children and adolescent. Our previous study confirmed that chronic PM2.5 exposure was positively associated with SBP, DBP and MAP, and increased the risk of the incidence of hypertension [17]. This conclusion was confirmed by several previous studies [46,47]. However, there were no study to show whether the decreased in ambient PM2.5 concentration has a protective effect on BP growth of children. Most of the current preliminary research have concentrated on artificially decreasing of indoor PM2.5 concentrations through air purification interventions [27,28,48,49]. However, the conclusions were inconsistent. In a randomized double-blind crossover trial among 35 healthy university students in Shanghai, China, BP decreased after a short-term indoor air purification intervention [28]. The concentration of PM2.5 decreased by 57% from 96.2 μg / m^3^ to 41.3 μg / m^3^, SBP and DBP decreased significantly by 2.7% and 4.8% respectively [28]. In our study, the exposure level of PM2.5 decreased 22.92 ± 4.51 μg / m^3^ from 2014 to 2019. This study examined the effect of changes in natural ambient PM2.5 concentrations on BP in children and adolescents using a prospective cohort study, without individual intervention, and the results were more convincing. Moreover, a randomized, double-blind crossover study in a Canadian First Nations community also confirmed that the use of air filter was associated with a decrease in systolic and diastolic BP [48]. In addition, this conclusion was identified by one study of older people in urban areas in the US [27]. However, for another Danish study, a two-week intervention using air filters had no effect on cardiovascular function in 51 non-smoking volunteers aged 51 and over [49]. The inconsistency results of these intervention studies may be due to the different genetic background, small sample size and the short duration of the intervention. The greening homes was associated with better vascular function, partly by mitigating the effects of ambient air pollution [50]. An intervention study of spontaneously hypertensive rats exposed to PM2.5 showed that BP reversibly recovered when PM2.5 exposure was stopped after a high exposure level, which confirmed the protective effect of PM2.5 reduction on BP levels [30].

In recent years, China has implemented a number of environmental protection policies [51], which have decreased ambient PM2.5 concentrations [52]. This has been confirmed by our research. In our study, PM2.5 concentration decreased from 65.01 ± 6.46 μg / m^3^ in 2014 to 42.08 ± 2.04 μg / m^3^ in 2019, which also far exceeded the WHO standards (10 μg / m^3^). This study found that a decrease in PM2.5 greater than 25.56 μg / m^3^ had the strongest protective effect on BP. The decrease in PM2.5 not only benefited the cardiovascular system, but also the respiratory system, pregnancy outcomes and clinical biochemical markers [29]. The findings of this study provided a theoretical basis for the development of environmental policies to improve the health of children. The improvement of air quality was crucial to the healthy development of children, and the whole society should continue to work hard to reduce ambient PM2.5 pollution.

The mechanisms by which PM2.5 induced hypertension are diverse, with inflammatory mechanisms predominating. Chronic exposure to PM2.5 led to an increase in sympathetic tone accompanied by an inflammatory response within the hypothalamic arcuate nucleus, increased expression of pro-inflammatory genes and activation of the inhibitor κB kinase [53] / nuclear factor-κB (NF-κB) pathway [54]. In addition, chronic exposure to PM2.5 also significantly increased the expression of pro-inflammatory cytokines in the lung, heart and hypothalamus, with significant increases in mRNA expression of TNFα, IL-6 and COX2. The hypertensive effects of PM2.5 exposure may be mediated by systemic inflammation, independent of lung inflammation. However, cessation of exposure alleviated inflammation in the heart and hypothalamus [30].

### Strengths and limitations

First, this study was based on a large cohort study of children, and it was the first time to illustrate the etiological relevance between the decreased PM2.5 level and BP growth and the IR of prehypertension / hypertension in children. Second, it examined the effect of decreased ecological ambient PM2.5 on BP level in children, and the findings are more generalizable. Third, this study adjusted for multiple variables and the results were more reliable. Fourth, the B*P* values were measured at least three times at three separated occasions, and the effect of decreased PM2.5 level on BP on each occasion was analysed, which may induce a more accurate result. In addition, there are two limitations in our study. First, this study was conducted in urban-rural areas of Chongqing with high concentration of PM2.5, the conclusion may be not applicable to areas with lower concentration of PM2.5 world widely. Second, only one air pollutant was analysed and the detail information about in-door pollutions was not included. However, combined concentration of PM2.5 around home and school addresses was calculated to represent the exposure level of each child, and passive smoking in-home was adjusted, which may close to true air pollution exposure levels.

## CONCLUSIONS

Our findings confirmed that a decreased level in PM2.5 was beneficial to BP level and the incidence of prehypertension and hypertension in children and adolescent, which may identify the etiological relevance between environmental protection and health effect of BP protection in early stage of life. However, the underlying mechanisms should be explored in further well-designed large sample size studies. Our study suggested that the government should implement the policies to decrease PM2.5 exposure levels in order to promote the well-being of children and adolescent.

## Additional material


Online Supplementary Document
