# Supplementary material for: The policy implemented by the government and the protection effect of PM2.5 decreasing on blood pressure in adolescents: From a quasi-experimental study
Source: J Glob Health. 2023 May 26;13:04050. doi: 10.7189/jogh.13.04050 (PMC10214769; doi:10.7189/jogh.13.04050)
Supplement: Online Supplementary Document [file jogh-13-04050-s001.pdf]

Supplementary table 1. The annual average exposure level of PM2.5 of included subjects (μg/m3)

Years	Total			Urban			Rural		
	Means of PM2.5	Min value	Max value	Means of PM2.5	Min value	Max value	Means of PM2.5	Min value	Max value
2000	48.16±6.35	35.4	55.19	53.93±1.13	36.81	55.19	42.07±3.02	35.4	54.58
2001	56.59±7.02	42.61	64.73	62.9±1.25	42.68	64.73	49.93±3.64	42.61	64.13
2002	56.97±7.8	41.71	65.88	63.94±1.32	41.93	65.65	49.6±4.18	41.71	65.88
2003	58.4±4.97	44.99	64.56	62.74±1.1	44.99	64.56	53.82±2.91	45.21	63.6
2004	60.78±5.85	47.17	68.1	65.87±1.15	47.17	68.1	55.4±3.57	47.39	67.54
2005	62.67±5.35	48.24	69.96	67.4±1.15	50.76	69.96	57.67±2.95	48.24	68.19
2006	64.26±6.08	48.68	71.67	69.62±1.18	49.47	71.67	58.61±3.51	48.68	71.5
2007	65.78±6.1	49.11	73.49	71.19±1.24	50.35	73.49	60.07±3.37	49.11	71.63
2008	66.07±5.55	50.61	72.2	71.03±1.09	51.59	72.2	60.83±2.9	50.61	71.28
2009	68.48±7.26	51.47	78.07	74.89±1.49	51.47	78.07	61.72±4.11	51.47	77.37
2010	70.05±7.34	51.47	79.65	76.61±1.42	51.75	79.65	63.12±3.9	51.47	77.18
2011	65.28±5.28	51.09	72.36	69.88±1.05	53.28	72.36	60.41±3.15	51.09	71.54
2012	64.56±5.15	51.22	72.33	69.11±1.06	52.12	72.33	59.76±2.93	51.22	70.76
2013	66.52±5.8	50.38	83.18	71.17±2.07	51.05	83.18	61.61±4.21	50.38	78.42
2014	64.18±7.56	47.06	75.08	70.94±1.36	49.38	75.08	57.05±4.07	47.06	72.06
2015	56.64±5.15	43.12	63.27	61.14±1.14	44.59	63.27	51.88±3.01	43.12	62.52
2016	55.69±6.17	41.63	62.87	61.25±1.24	42.47	62.87	49.81±3.06	41.63	62.17
2017	52.34±2.47	42.18	55.12	54.22±0.91	43.39	55.12	50.36±2	42.18	54.83
2018	43.02±1.99	35.51	48.82	44.45±0.88	36.57	47.29	41.52±1.71	35.51	48.82
2019	41.83±2.28	30.19	44.42	43.79±0.73	30.19	44.42	39.77±1.36	32	44.42

Supplementary Table 2 The effect of the decreased level of PM2.5 by P_{50}(25.56 μg/m^3) on blood pressure value

Dependent variables	Models	Decreased PM2.5 level< 25.56 μg/m^3	Decreased PM2.5 level≥25.56 μg/m^3	The difference of BP values	P
**SBP, mmHg**					
	**Model 1**	107.060(106.5,107.60)	103.900(103.4,104.40)	-3.153(-3.87,-2.43)	<0.001
	**Model 2**	107.190(106.7,107.60)	103.760(103.3,104.20)	-3.422(-4.05,-2.80)	<0.001
	**Model 3**	107.150(106.6,107.70)	103.790(103.3,104.30)	-3.356(-4.09,-2.62)	<0.001
	**Model 4**	107.250(106.7,107.80)	103.710(103.2,104.20)	-3.538(-4.29,-2.79)	<0.001
**DBP, mmHg**					
	**Model 1**	63.529(63.15,63.90)	61.539(61.17,61.91)	-1.990(-2.52,-1.46)	<0.001
	**Model 2**	63.474(63.11,63.84)	61.569(61.21,61.93)	-1.904(-2.42,-1.39)	<0.001
	**Model 3**	63.432(63.00,63.86)	61.614(61.22,62.01)	-1.818(-2.43,-1.21)	<0.001
	**Model 4**	63.455(63.02,63.89)	61.594(61.19,62.00)	-1.860(-2.49,-1.23)	<0.001
**MAP, mmHg**					
	**Model 1**	78.038(77.66,78.42)	75.660(75.29,76.03)	-2.377(-2.91,-1.85)	<0.001
	**Model 2**	78.044(77.69,78.40)	75.634(75.29,75.98)	-2.410(-2.91,-1.91)	<0.001
	**Model 3**	78.004(77.59,78.42)	75.673(75.29,76.06)	-2.331(-2.92,-1.74)	<0.001
	**Model 4**	78.052(77.63,78.47)	75.632(75.25,76.02)	-2.420(-3.02,-1.82)	<0.001
**Difference of SBP, mmHg**					
	**Model 1**	10.048(9.507,10.59)	6.695(6.166,7.225)	-3.353(-4.11,-2.60)	<0.001
	**Model 2**	10.117(9.600,10.63)	6.611(6.106,7.117)	-3.505(-4.24,-2.77)	<0.001
	**Model 3**	10.074(9.477,10.67)	6.673(6.119,7.227)	-3.402(-4.26,-2.55)	<0.001
	**Model 4**	10.179(9.574,10.79)	6.581(6.021,7.141)	-3.598(-4.47,-2.72)	<0.001
**Difference of DBP, mmHg**					
	**Model 1**	4.349(3.911,4.79)	1.990(1.562,2.42)	-2.358(-2.97,-1.75)	<0.001
	**Model 2**	4.249(3.812,4.69)	2.056(1.629,2.482)	-2.193(-2.81,-1.58)	<0.001
	**Model 3**	4.164(3.657,4.67)	2.170(1.701,2.639)	-1.994(-2.72,-1.27)	<0.001
	**Model 4**	4.195(3.680,4.71)	2.143(1.666,2.619)	-2.052(-2.80,-1.31)	<0.001
**Difference of MAP, mmHg**					
	**Model 1**	6.249(5.830,6.667)	3.559(3.149,3.968)	-2.690(-3.28,-2.10)	<0.001
	**Model 2**	6.205(5.794,6.615)	3.574(3.173,3.976)	-2.630(-3.21,-2.05)	<0.001
	**Model 3**	6.134(5.659,6.609)	3.671(3.230,4.112)	-2.463(-3.14,-1.78)	<0.001
	**Model 4**	6.190(5.707,6.673)	3.622(3.175,4.069)	-2.568(-3.27,-1.87)	<0.001

Model 1: The crude model

Model 2: adjusted age, sex, height and weight

Model 3: age, sex, height, weight, maternal education, puberty development, passive smoking, maternal obesity, birthweight, household income, vegetable intake, red meat intake, pickle intake, sleep quality and physical activity

Model 4: age, sex, height, weight, maternal education, puberty development, passive smoking, maternal obesity, birthweight, household income, vegetable intake, red meat intake, pickle intake, sleep quality, physical activity, personality character, FBG, insulin, creatinine, TG and HDL

Supplementary Table 3. The effect of 1μg/m3 decrease of the PM2.5 on BP value using mixed model to adjust the non-independence within each cluster

	Model 1		Model 2		Model 3		Model 4	
**Dependent variables**	**β(95%CIs)**	**P**	**β(95%CIs)**	**P**	**β(95%CIs)**	**P**	**β(95%CIs)**	**P**
**BP value**								
SBP, mmHg	-0.59(-0.74, -0.44)	<0.001	-0.46(-0.60, -0.31)	<0.001	-0.69(-0.96, -0.42)	<0.001	-1.33(-1.72, -0.93)	<0.001
DBP, mmHg	-0.37(-0.47, -0.26)	<0.001	-0.31(-0.42, -0.20)	<0.001	-0.41(-0.61, -0.21)	<0.001	-0.74(-1.04, -0.45)	<0.001
MAP, mmHg	0.19(-0.13, 0.50)	0.2408	-0.36(-0.47, -0.25)	<0.001	-0.50(-0.70, -0.30)	<0.001	-0.94(-1.23, -0.64)	<0.001
**The difference of BP values**								
Different of SBP, mmHg	-0.63(-0.79, -0.48)	<0.001	-0.56(-0.71, -0.40)	<0.001	-0.66(-0.94, -0.38)	<0.001	-1.24(-1.65, -0.83)	<0.001
Different of DBP, mmHg	-0.43(-0.56, -0.31)	<0.001	-0.41(-0.53, -0.28)	<0.001	-0.38(-0.61, -0.15)	0.001	-0.62(-0.95, -0.29)	0.0002
Different of MAP, mmHg	-0.50(-0.62, -0.38)	<0.001	-0.46(-0.58, -0.34)	<0.001	-0.48(-0.69, -0.26)	<0.001	-0.83(-1.14, -0.52)	<0.001

Model 1: The crude model

Model 2: adjusted age, sex, height and weight

Model 3: adjusted age, sex, height, weight, maternal education, puberty development, passive smoking, maternal obesity, birthweight, household income, vegetable intake, red meat intake, pickle intake, sleep quality and physical activity

Model 4: adjusted age, sex, height, weight, maternal education, puberty development, passive smoking, maternal obesity, birthweight, household income, vegetable intake, red meat intake, pickle intake, sleep quality, physical activity, personality character, FBG, insulin, creatinine, TG and HDL

Supplementary Table 4 The effect of the decreased level of PM2.5 by P_{50} (25.56 μg/m^3) on prehypertension or hypertension

	Model 1		Model 2		Model 3		Model 4	
**Dependent variables**	**RR(95%CIs)**	**P**	**RR(95%CIs)**	**P**	**RR(95%CIs)**	**P**	**RR(95%CIs)**	**P**
**Prehypertension or hypertension**								
First occasion	0.706(0.565,0.883)	0.002	0.713(0.570,0.892)	0.003	0.749(0.572,0.980)	0.035	0.708(0.538,0.931)	0.014
Second occasion	0.407(0.251,0.659)	<0.001	0.408(0.253,0.659)	<0.001	0.357(0.205,0.622)	<0.001	0.304(0.173,0.534)	<0.001
Third occasion	0.404(0.201,0.814)	0.011	0.383(0.192,0.766)	0.007	0.315(0.137,0.722)	0.006	0.256(0.109,0.600)	0.002
**Systolic prehypertension or hypertension**								
First occasion	0.660(0.507,0.860)	0.002	0.654(0.502,0.852)	0.002	0.709(0.513,0.980)	0.037	0.643(0.463,0.894)	0.009
Second occasion	0.350(0.195,0.628)	<0.001	0.354(0.198,0.632)	<0.001	0.253(0.129,0.499)	<0.001	0.221(0.109,0.446)	<0.001
Third occasion	0.553(0.264,1.157)	0.116	0.521(0.251,1.082)	0.081	0.446(0.186,1.069)	0.070	0.366(0.148,0.901)	0.029
**Diastolic prehypertension or hypertension**								
First occasion	0.700(0.494,0.992)	0.045	0.707(0.495,1.009)	0.056	0.707(0.468,1.067)	0.099	0.688(0.454,1.043)	0.079
Second occasion	0.494(0.266,0.917)	0.025	0.476(0.258,0.880)	0.018	0.503(0.253,1.000)	0.050	0.401(0.195,0.825)	0.013
Third occasion	0.265(0.099,0.713)	0.008	0.249(0.093,0.664)	0.006	0.143(0.039,0.531)	0.004	0.099(0.024,0.406)	0.001

Model 1: the crude model

Model 2: adjusted age, sex, height and weight

Model 3: adjusted age, sex, height, weight, maternal education, puberty development, passive smoking, maternal obesity, birthweight, household income, vegetable intake, red meat intake, pickle intake, sleep quality and physical activity

Model 4: adjusted age, sex, height, weight, maternal education, puberty development, passive smoking, maternal obesity, birthweight, household income, vegetable intake, red meat intake, pickle intake, sleep quality, physical activity, personality character, FBG, insulin, creatinine, TG and HDL

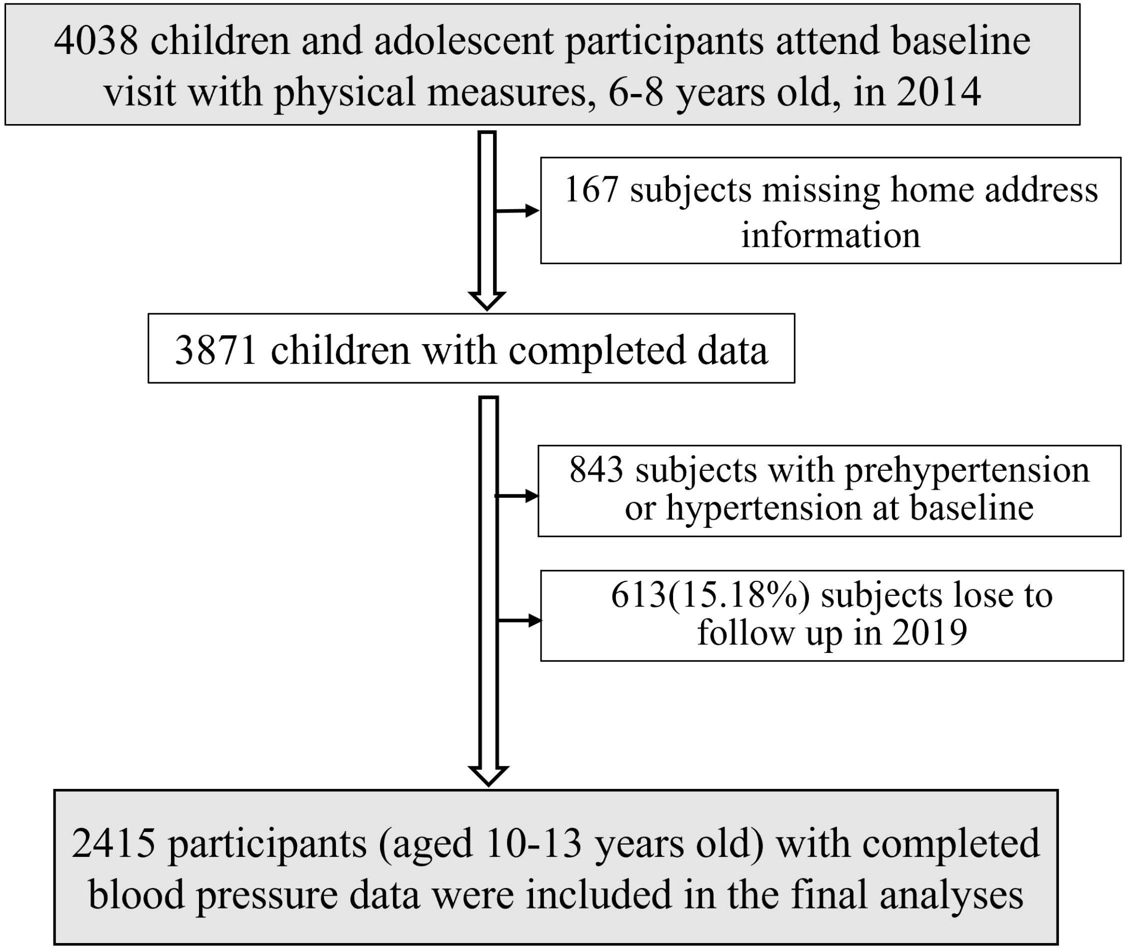

Supplementary figure 1. The flow chart of the included participants
